# Decompressive craniectomy in traumatic brain injury: insights from a 15-year multicentre cohort in Sweden

**DOI:** 10.1186/s13049-026-01585-6

**Published:** 2026-02-26

**Authors:** Francisco Leal-Méndez, Klas Holmgren, Alba Corell, Tijana Nesic, Peter Lindvall, Bjartur Sæmundsson, Robert Nilsson, Per Enblad, Fartein Velle, Anders Lewén, Richard Ågren, Alexander Fletcher-Sandersjöö, Teodor Svedung-Wettervik

**Affiliations:** 1 Department of Medical Sciences, Section of Neurosurgery, Uppsala University, Uppsala, 751 85 Sweden; 2 Department of Clinical Sciences - Neurosciences, Umeå University, Umeå, Sweden; 3 Department of Clinical Neuroscience, Institute of Neuroscience and Physiology at the Sahlgrenska Academy, University of Gothenburg, Gothenburg, Sweden; 4 Department of Neurosurgery, Sahlgrenska University Hospital, Gothenburg, Sweden; 5 Department of Neurosurgery, Karolinska University Hospital, Stockholm, Sweden; 6 Department of Clinical Neuroscience, Karolinska Institutet, Stockholm, Sweden; 7 Department of Physiology and Pharmacology, Karolinska Institutet, Stockholm, Sweden

**Keywords:** Decompressive craniectomy, Neurointensive care, Outcome, Traumatic brain injury, Mortality

## Abstract

**Background:**

Decompressive craniectomy (DC) is a last-resort treatment for severe traumatic brain injury (TBI) with refractory intracranial hypertension. Randomized controlled trials (RCTs) report mixed and sometimes conflicting results, leaving uncertainties regarding indications, timing, and long-term benefits. This study explored DC practices and outcomes in a contemporary Swedish setting contextualised in modern RCT evidence.

**Methods:**

This retrospective multicentre study included 299 TBI patients who underwent DC between 2008 and 2022 across four Swedish neurosurgical centres. Clinical, radiological, surgical, and outcome data (6-months Glasgow Outcome Scale) were collected. Differences across centres and between adults/children were analysed.

**Results:**

Annual DC rate remained stable over 15 years, modestly declining from 3.6 to 3.2 per million inhabitants. Significant regional differences were observed in timing, indications, and techniques. Proportion of primary versus secondary DC and surgery timing remained unchanged, though bifrontal DC decreased. Patients were young (median age 37), predominantly male (76%), severely injured (GCS M < 6), and 48% had unreactive pupils. Radiological improvement in mass effect post-DC (midline shift, basal cisterns) was significant (*p* < 0.001). Re-operation for haemorrhage occurred in 10%, complementary decompression, surgical-site infection, and subdural hygroma each occurred in ~ 5%. At 6 months, 60% had unfavourable outcomes and 11% were deceased. Higher age, lower GCS, comorbidities, impaired pupillary reactivity and obliterated basal cisterns independently predicted unfavourable outcome.

**Conclusions:**

Landmark RCTs appear to have had limited influence on Swedish DC practice, which remains variable across centres. Real-world outcomes were more favourable than in recent RCTs and other acute brain injuries.

**Supplementary Information:**

The online version contains supplementary material available at 10.1186/s13049-026-01585-6.

## Introduction

Traumatic brain injury (TBI) remains one of the leading causes of death and long-term disability worldwide [1]. In severe TBI, elevated intracranial pressure (ICP) is a relatively common complication, contributing to secondary brain injury and poor outcome [2, 3]. Initial management focuses on basic ICP-lowering strategies, including head-of-bed elevation, mild hyperventilation, osmotherapy (e.g., mannitol/hypertonic saline), sedation (including barbiturates in selected cases), and surgical evacuation of space-occupying intracranial lesions [3–8]. When these measures fail, secondary decompressive craniectomy (DC) may be performed to prevent brain herniation and alleviate intracranial hypertension. DC can also be performed as a primary procedure, during evacuation of a traumatic lesion when intraoperative brain swelling prevents bone flap reimplantation. The decision to perform DC is highly complex, requiring careful consideration of timing and weighing of risks and benefits, including long-term outcomes [6, 7, 9–15]. While DC can provide immediate control of refractory ICP, it carries risks of postoperative bleeding, infection, and disturbances in cerebrospinal fluid circulation [14, 16]. A subsequent cranioplasty is also necessary, adding further risks to account for, such as postoperative haemorrhage, infection, and bone flap resorption [17, 18]. DC should thus only be considered after exhausting less invasive options; however not to the extent that allows further secondary brain injury to develop [6]. Moreover, outcome prognostication is difficult, raising concerns that DC may prolong survival without meaningful neurological recovery in several cases [19].

In this context and modern era of evidence-based medicine, several randomised controlled trials (RCTs) have sought to clarify the role of DC in TBI. The DECRA trial investigated early, secondary bilateral DC for ICP > 20 mmHg for 15 min during an hour during the first 72 h and found that functional outcomes were inferior to patients receiving maximal medical management [9]. On the contrary, the RESCUE-ICP trial, employed a higher ICP threshold (> 25 mmHg for ≥ 1 h), demonstrated reduced mortality, however increased severe disability rates at 6 months [9] with a modest shift towards more favourable outcomes at 24 months [20]. The recent RESCUE-ASDH trial examined whether evacuation of an acute subdural haematoma (ASDH) should also include primary DC in cases of clinical equipoise, arriving at similar functional outcomes and fewer wound complications with craniotomy alone, however an increased need for additional surgeries [10]. Furthermore, improved outcomes in paediatric TBI patients have been demonstrated with secondary bi-temporal DC, although evidence remains limited to a pilot RCT [21].

Despite level I evidence from these RCTs, translating their findings into clinical practice remains challenging [15, 22, 23]. For example, DECRA has been criticized for intervening too early, rather risking overtreatment and complications than benefitting from meaningful ICP reduction, whereas in RESCUE-ICP, DC may have been postponed to a stage when substantial secondary injury had already reached an unsalvageable state [9, 11, 15, 22, 23]. Both trials also had significant crossover, with DC often performed after failure of maximal medical therapy including barbiturates in the control groups [9, 11, 24]. Moreover, strict inclusion criteria also limit the external validity, of these RCTs, as real-world patients frequently fall outside the trial populations [9, 11, 15, 22, 23]. As such, they may fail to capture the clinically perceived optimal window for intervention – one that is often determined by clinical judgment in the context of the complex and evolving nature of TBI and in conjunction with other second- and third-tier ICP-lowering therapies [15, 22, 23, 25]. This raises doubts about the RCTs applicability to current practice, as they largely examine the extremes of timing rather than the nuanced decisions faced in daily care. Consequently, treatment is often left to the discretion of each neurosurgical institution and, ultimately, the neurosurgeon on-call, which inevitably results in substantial variability across centres in indications, timing, surgical technique (e.g., hemicraniectomy vs. bi-frontal), and long-term outcomes.

Thus, in the era of modern medicine driven by RCTs, we aimed to characterize real-world TBI patients treated with DC in a contemporary Swedish multicentre context. Specifically, we sought to describe temporal trends in DC use, patient characteristics, timing of surgery, surgical techniques, complication rates, and functional outcomes. A particular focus was to explore differences across neurosurgical centres and between specific patient populations, such as adults versus paediatric patients. We hypothesized that the overall rate and type of DC would remain relatively stable over time, despite the publications of key RCTs during this period. However, we also hypothesised a presence of considerable variation in patient selection across centres, potentially influencing both complication rates and functional outcomes. Paediatric TBI cases treated with DC were expected to be rare, but likely to involve different patient profiles and slightly more favourable outcomes.

## Materials and methods

### Patients and study design

This retrospective, multicentre observational study included all 299 patients who underwent DC for TBI between 2008 and 2022 at the neurosurgical departments in Stockholm, Uppsala, Umeå, and Gothenburg (2008–2018 only). Together, these centres serve a catchment population of approximately 7.5 million, representing about 72% of the Swedish population. Patients aged 18 years or older were classified as adults, while those younger than 18 were defined as children.

### Healthcare structure and general management

In the Swedish healthcare system, patients with moderate-to-severe TBI are in general initially managed at their nearest hospital, local or university, according to Advanced Trauma Life Support (ATLS) principles [26]. Unconscious patients requiring intubation and mechanical ventilation and/or those with intracranial mass lesions are typically transferred to the neurosurgical department at the university hospital serving the respective catchment area, where they are admitted to the NIC unit for monitoring. Life-saving evacuations of extracerebral haematomas are in a small subset of patients performed by general surgeons at the local hospital before transportation to the neurosurgical department, in case of mass lesions and brain herniation syndrome [27]. General management follows the Brain Trauma Foundation guidelines [4, 28], with local adaptations [3, 12, 16, 29, 30]. In the university hospitals, ICP monitoring is routinely initiated in unconscious patients, using external ventricular drains (EVD) or intraparenchymal probes. ICP levels above 20 mmHg are actively avoided. Standard ICP-lowering measures include head elevation, hyperosmolar therapy, mild hyperventilation, and cerebrospinal fluid drainage via an EVD when applicable. In cases of refractory intracranial hypertension, barbiturates and DC are considered last-tier interventions [6]. DC is performed either as a primary intervention, early and without preceding ICP monitoring, in the presence of extensive traumatic intracranial lesions with significant midline shift or if bone flap reimplantation appears inadvisable during primary surgery, while otherwise as a secondary rescue procedure in cases of medically refractory intracranial hypertension. Unilateral hemicraniectomy is the preferred technique for lateralized mass lesions or unilateral brain oedema, whereas bi-frontal DC is generally reserved for bilateral oedema without clear lateralization. The primary goal of all DC procedures is maximal decompression, achieved through removal of a large bone flap combined with duraplasty.

### Data acquisition

Clinical, radiological, and outcome data were retrospectively extracted from electronic medical records and neuroimaging archives. Demographic variables included age and sex. The Charlson Comorbidity Index (CCI), a validated tool for quantifying comorbid disease burden, was also analysed [31]. The severity of the primary brain injury was assessed based on clinical (Glasgow Coma Scale Motor [GCS M] [32] score) and pupillary reactivity at admission to the NIC and radiological (Marshall grade) variables. Treatment variables included type of ICP-monitoring method (EVD, intraparenchymal probe, both or no measure), evacuation of intracranial haemorrhages, and barbiturate coma. Furthermore, detailed DC-related data were collected, including timing (days post-injury), indication (primary/secondary), type (uni-/bilateral), and area (cm^2^). The latter was quantified by measuring multiple anteroposterior diameters (tabula externa) and craniocaudal height (slice thickness) of the cranial defects on the postoperative computed tomography (CT) scan), calculating the total decompression area (in cm^2^), as described in a previous study [14]. Midline shift and extent of basal cistern compression (open/compressed/obliterated) were assessed on the CT scan immediately prior to, and after, DC. Expected mortality and unfavourable outcome were calculated for adults (≥ 18 years) using the IMPACT core model (age, motor component of the GCS and pupil reactivity) (http://www.tbi-impact.org/?p=impact/calc#calcresults).

Postoperative DC complications were also noted, including re-operation due to postoperative haemorrhage, complementary bony decompression, and surgical-site infections. In addition, cerebrospinal fluid circulation (CSF) disturbances requiring shunt insertion were documented.

Functional outcomes were assessed at approximately six months post-DC using the Glasgow Outcome Scale (GOS) [33]. Outcomes were dichotomized as favourable (GOS 4–5) or unfavourable (GOS 1–3) and survival (GOS 2–5) or mortality (GOS 1). Assessments were performed by 1–3 neurosurgical trainees per centre, all trained in retrospective outcome evaluation. Information was obtained from medical records, outpatient visits, telephone follow-ups, and hospital discharge summaries. In cases of insufficient data, outcome was reported as missing.

### Statistical analysis

Statistical analyses were performed using R software (RStudio version 2024.04.1). Continuous and ordinal variables were summarized as medians with interquartile ranges (IQR), while categorical variables were reported as counts and percentages. We first explored differences between centres and between patient populations (adult vs. paediatric) with regards to demographics, injury severity, management, DC-related variables, and outcomes. The between-group comparisons were performed using Kruskal–Wallis test (inter-centre comparisons) or Mann–Whitney U test (adult vs. paediatric) for continuous variables and Chi-square or Fisher’s exact tests for categorical variables, as appropriate. Wilcoxon signed rank test was used to evaluate paired pre- and postoperative midline shift. Paired changes in basal cistern status were assessed with Bowker’s test of symmetry. Univariate logistic regressions were conducted to assess the association between demographic, clinical, and radiological descriptors of primary brain injury, as well as DC-related variables, with unfavourable outcome and mortality, respectively. The independent variables significantly associated with each dependent variable in the univariate regressions were thereafter entered into multivariable logistic regression models for evaluating the same outcomes. Statistical significance was defined as *p*-values < 0.05.

### Ethics

The study was approved by the Swedish Ethical Review Authority (Dnr: 2023–02347-01, decision date: 2023–05–17). Informed consent was waived.

## Results

### Patient characteristics – entire cohort, differences among centres, and adult/paediatric cohorts

In the entire cohort comprising 299 TBI patients treated with DC (Tables 1 and 2), the median age was 37 (IQR 23–52) years, and the majority were male (76%). Twenty-three percent exhibited a CCI score of 1 or higher. At admission, the median GCS M score was 4 (IQR 2–5) and 48% of patients had at least one unreactive pupil. Most patients (81%) had Marshall classification score of V-VI, indicating presence mass lesions. ICP-monitoring was utilized in nearly all cases (99%), predominantly by means of an intraparenchymal probe alone (51%) or in combination with EVD (34%). Barbiturates were administered in 55% of cases.

**Table 1:** DC utilisation over the different 5-year periods – rate, timing, indication, and type of DC

Period	Variable	Entire	Stockholm	Gothenburg^a^	Uppsala	Umeå
‍‍2008-2012	DC Cases per period, n	112	31	23	29	29
	DC Cases/Year, n	22.4	6.2	4.6	5.8	5.8
	DC Cases/Million inhabitants/Year, n^b^	3.6	3.0	2.7	3.2	8.1
	Time from trauma to DC (days), median (IQR)	1 (1–2)	1 (1–2)	1 (1–2)	1 (1–3)	1 (1–2)
	Indication for DC (primary/secondary), n (%)	57/55 (51/49%)	17/14 (55/45%)	14/9 (61/39%)	15/14 (52/48%)	11/18 (38/62%)
	Type of DC (hemi/bifrontal), n (%)	98/14 (88/12%)	30/1 (97/3%)	17/6 (74/26%)	28/1 (97/3%)	23/6 (79/21%)
‍‍2013-2017	DC Cases per period, n	106	31	27	25	23
	DC Cases/Year, n	21.2	6.2	5.4	5.0	4.6
	DC Cases/Million inhabitants/Year, n^b^	3.2	3.0	3.0	2.6	5.7
	Time from trauma to DC (days), median (IQR)	1 (1–3)	1 (1–4)	1(1–2)	2 (1–6)	2 (1–3)
	Indication for DC (primary/secondary), n (%)	46/60 (43/57%)	15/16 (48/52%)	16/11 (59/41%)	8/17 (32/68%)	7/16 (30/70%)
	Type of DC (hemi/bifrontal), n (%)	90/16 (85/15%)	30/1 (97/3%)	23/4 (85/15%)	19/6 (76/24%)	18/5 (78/22%)
‍‍2018-2022	DC Cases per period, n	81	20	NA	35	26
	DC Cases/Year, n	16.2	4.0	NA	7.0	5.2
	DC Cases/Million inhabitants/Year, n^b^	3.2	1.8	NA	3.5	5.8
	Time from trauma to DC (days), median (IQR)	1 (1–3)	1 (1–3)	NA	1 (1–4)	1 (1–2)
	Indication for DC (primary/secondary), n (%)	39/42 (48/52%)	8/12 (40/60%)	NA	21/14 (60/40%)	10/16 (38/62%)
	Type of DC (hemi/bifrontal), n (%)	77/4 (95/5%)	20/0 (100/0%)	NA	33/2 (94/6%)	24/2 (92/8%)
2008–2022 (Total Period)	DC Cases per period, n	299	82	50	89	78
	DC Cases/Year, n	19.9	5.5	5.0	5.9	5.2
	DC Cases/Million inhabitants/Year, n^b^	3.0	2.6	2.9	3.1	6.4

*Abbreviations: DC *Decompressive Craniectomy, *NA *Not Applicable

^a^Gothenburg has no data for period 2018–2022

^b^Population number obtained from SCB (Swedish National Statistics Agency) and averaged for respective year period

**Table 2:** Demographics, admission variables, clinical course, and functional outcome – entire cohort and individual centres

Variables	Entire Cohort	Stockholm	Gothenburg	Uppsala	Umeå	*p*-value
Patients, n (%)	299 (100%)	82 (27%)	50 (17%)	89 (30%)	78 (26%)	NA
Age (years), median (IQR)	37 (23–52)	32 (23–47)	38 (24–52)	42 (24–53)	34 (21–56)	0.38
Sex (male/female), n (%)	228/71 (76/24%)	64/18 (78/22%)	40/10 (80/20%)	67/22 (75/25%)	57/21 (73/27%)	0.80
CCI Score, n (%)						
0	228 (77%)	65 (81%)	30 (60%)	75 (84%)	58 (74%)	***0.019***
≥ 1	69 (23%)	15 (19%)	20 (40%)	14 (16%)	20 (26%)	
GCS M at admission, median (IQR)	4 (2–5)	3 (1–5)	6 (5–6)	5 (3–5)	4 (2–5)	**< *0.001***
Pupillary Reaction at admission, n (%)						
*Normal, n (%)*	148 (52%)	31 (40%)	23 (46%)	62 (71%)	32 (46%)	**< *0.001***
*1 Unreactive, n (%)*	75 (26%)	30 (38%)	11 (22%)	16 (18%)	18 (26%)	
*2 Unreactive, n (%)*	62 (22%)	17 (22%)	16 (32%)	10 (11%)	19 (28%)	
Marshall Classification, n (%)						
*I-II, n (%)*	34 (11%)	2 (2%)	0 (0%)	27 (30%)	5 (6%)	**< *0.001***
*III-IV, n (%)*	24 (8%)	5 (6%)	0 (0%)	10 (11%)	9 (12%)	
*V-VI, n (%)*	241 (81%)	75 (92%)	50 (100%)	52 (59%)	64 (82%)	
ICP-monitoring, n (%)						
*No ICP-monitoring*	3 (1%)	2 (2%)	1 (2%)	0 (0%)	0 (0%)	**< *0.001***
*EVD*	42 (14%)	20 (24%)	9 (18%)	7 (8%)	6 (8%)	
*Intraparenchymal*	153 (51%)	33 (40%)	28 (56%)	57 (64%)	35 (45%)	
*Both*	101 (34%)	27 (34%)	12 (24%)	25 (28%)	37 (47%)	
Intracranial hematoma evacuation (yes), n (%)	241 (81%)	75 (91%)	43 (86%)	65 (73%)	58 (74%)	***0.006***
Barbiturates (yes), n (%)	159 (55%)	43 (52%)	33 (67%)	29 (33%)	54 (79%)	**< *0.001***
Unfavourable Outcome^a^, n (%)	170 (60%)	47 (61%)	24 (49%)	45 (55%)	54 (72%)	***0.049***
Mortality, n (%)	33 (11%)	12 (15%)	4 (8%)	4 (5%)	13 (17%)	***0.047***

**Missing data:** Age = 1; GCS M = 47; CCI = 2; Pupillary Reaction = 14; Barbiturates = 11; Unfavourable Outcome = 15; Mortality = 15

Bold and italics represent statistical significance

*Abbreviations: GCS M *Glasgow Coma Scale Motor, *CCI *Charlson Co-morbidity Index, *GOS* Glasgow Outcome Scale, *NA* Not applicable

^a^Unfavourable Outcome = GOS 1–3

Some differences were observed in these variables amongst the participating centres (Table 2). For example, the rate of impaired pupillary reactivity was significantly (*p* < 0.001) more frequent in Stockholm, Gothenburg and Umeå (60%, 54% and 54% respectively) compared to Uppsala (29%). CCI ≥ 1 was also notably higher (*p* = 0.019) in Gothenburg compared with Stockholm (19%), Uppsala (16%) and Umeå (26%).

Paediatric patients treated with DC were rare (*n* = 37 [12%]). Compared to adults, children were marginally more often female (38% vs. 22%; *p* = 0.053), had a lower comorbidity burden (CCI ≥ 1 in 8% vs. 75%; *p* < 0.001), and slightly less frequently underwent haematoma evacuation (70% vs. 82%; *p* = 0.092). The exclusive paediatric and adult cohorts are also presented separately for each centre in Supplementary Tables 2 and 3.

### DC rate, regional variations and temporal trends

**Fig. 1:**
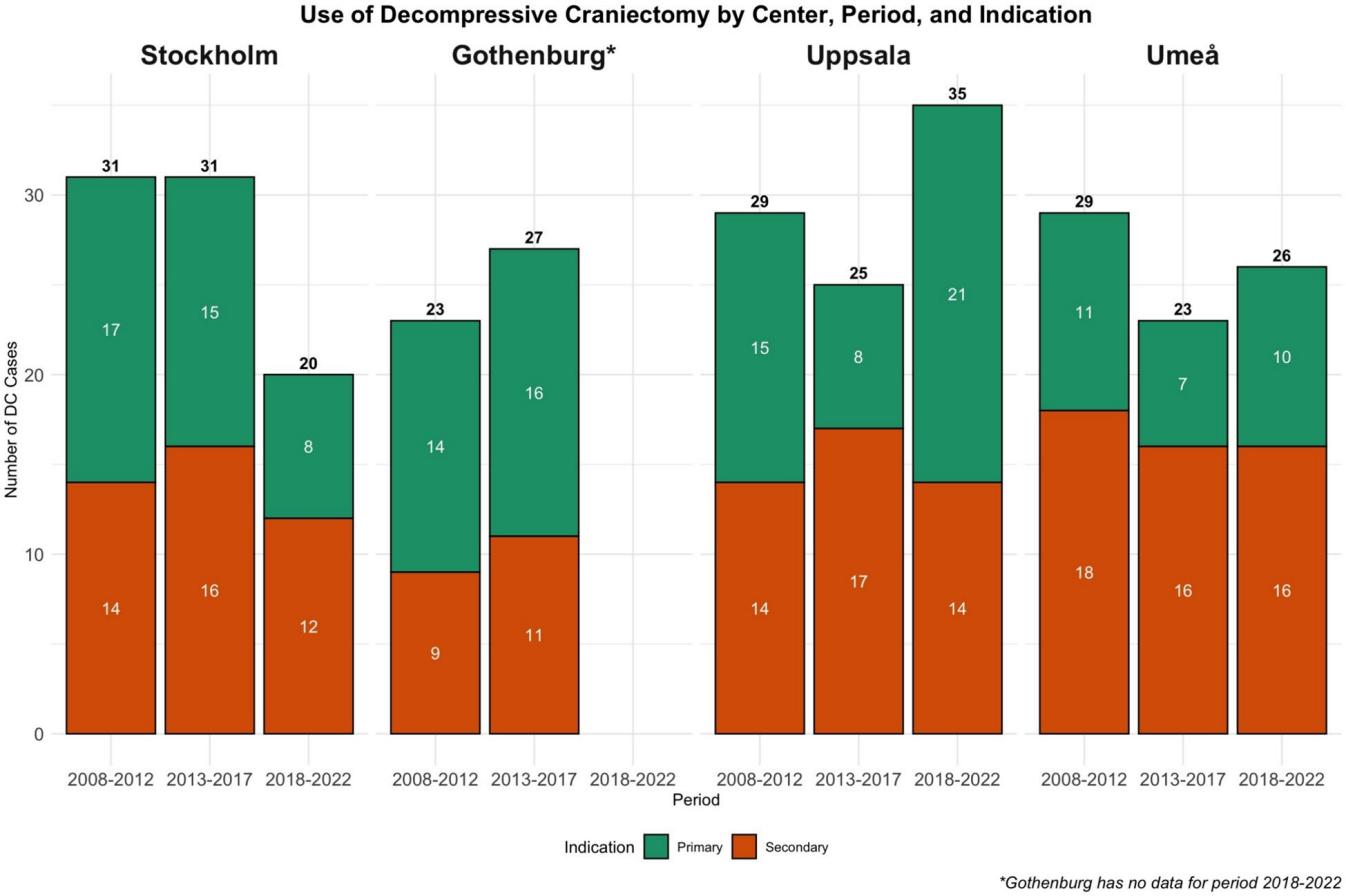
Use of Decompressive Craniectomy over 5-Year Periods per Centre

As shown in Table 1 and illustrated in Fig. 1, the overall annual DC rate was 19.9 and remained stable with a modest decline over the three five-year periods, from 3.6 per million inhabitants and year in 2008–2012 to 3.2 per million inhabitants and year, both in 2013–2017 and 2018–2022. The temporal trend in DC use varied across centres. For example, Stockholm showed a modest decline from the first to the last period, while Uppsala experienced a mid-period dip followed by a resurgence in 2018–2022. Notably, Umeå consistently had a higher DC rate per-capita (5.7–8.1 per million inhabitants and year) compared to the other centres, which remained within 1.8–3.6 per million inhabitants and year. As shown in Fig. 1, the number and proportion of primary versus secondary DC remained relatively consistent across centres and throughout the three five-year periods. Similarly, the timing of DC was largely stable, predominantly occurring within the first 72 h post-injury. However, the relative frequency of bi-frontal DC declined from 15 to 5% in the most recent period.

### DC – indication, timing, type and complications

The median time point of DC surgery was the first day post-injury (IQR 1–3 days; Table 3), with 47% of patients undergoing primary DC and 53% secondary DC for refractory intracranial hypertension. Unilateral hemicraniectomy was the predominant approach (89%), while bi-frontal DC was rare (11%). The median bone-flap area was 98 cm^2^ with no significant statistical difference between centres (IQR 84–110; *p* = 0.268).

**Table 3:** DC surgery: timing, indication, and complications – entire cohort and individual centres

Variables	Entire Cohort	Stockholm	Gothenburg	Uppsala	Umeå	*p*-value
Time from trauma to DC (days), median (IQR)	1 (1–3)	2 (1–3)	1 (1–2)	2 (1–4)	1 (1–2)	** < *0.001***
Indication for DC (primary/secondary), n (%)	142/157 (47/53%)	40/42 (49/51%)	30/20 (60/40%)	44/45 (49/51%)	28/50 (36/64%)	0.056
Type of DC (hemi/bifrontal), n (%)	265/34 (89/11%)	80/2 (98/2%)	40/10 (80/20%)	80/9 (90/10%)	65/13 (83/17%)	***0.005***
DC size (cm^2^), median (IQR)	97.7 (83.8–109.8)	97.4 (88.4–107.9)	92.0 (72.0–107.1)	99.6 (86.3–118.0)	97.4 (83.4–107.2)	0.268
Reoperation due to Post-DC bleeding (yes), n (%)^a^	29 (10%)	8 (10%)	8 (16%)	6 (7%)	7 (9%)	0.361
Post-DC extension of bony decompression, n (%)	17 (6%)	0 (0%)	5 (10%)	5 (6%)	7 (9%)	***0.041***
Post-DC surgical site-infection, n (%)	11 (4%)	1 (1%)	6 (12%)	3 (3%)	1 (1%)	***0.006***
Post-DC subdural hygroma, n (%)	12 (4%)	3 (4%)	1 (2%)	4 (5%)	4 (5%)	0.836
Post-DC VP-shunt, n (%)	36 (12%)	8 (10%)	9 (18%)	12 (13%)	7 (9%)	0.399

**Missing Data:** DC Size (in cm^2^) = 11

Bold and italics represent statistical significance

*Abbreviations: DC* Decompressive Craniectomy, *VP-shunt* Ventriculoperitoneal shunt

^a^Type of bleeding that was evacuated; Acute Subdural Hematoma or/and Epidural Hematoma = Ipsilateral: 16/Contralateral: 8; Contusions = Ipsilateral: 4/Contralateral: 1

**Fig. 2:**
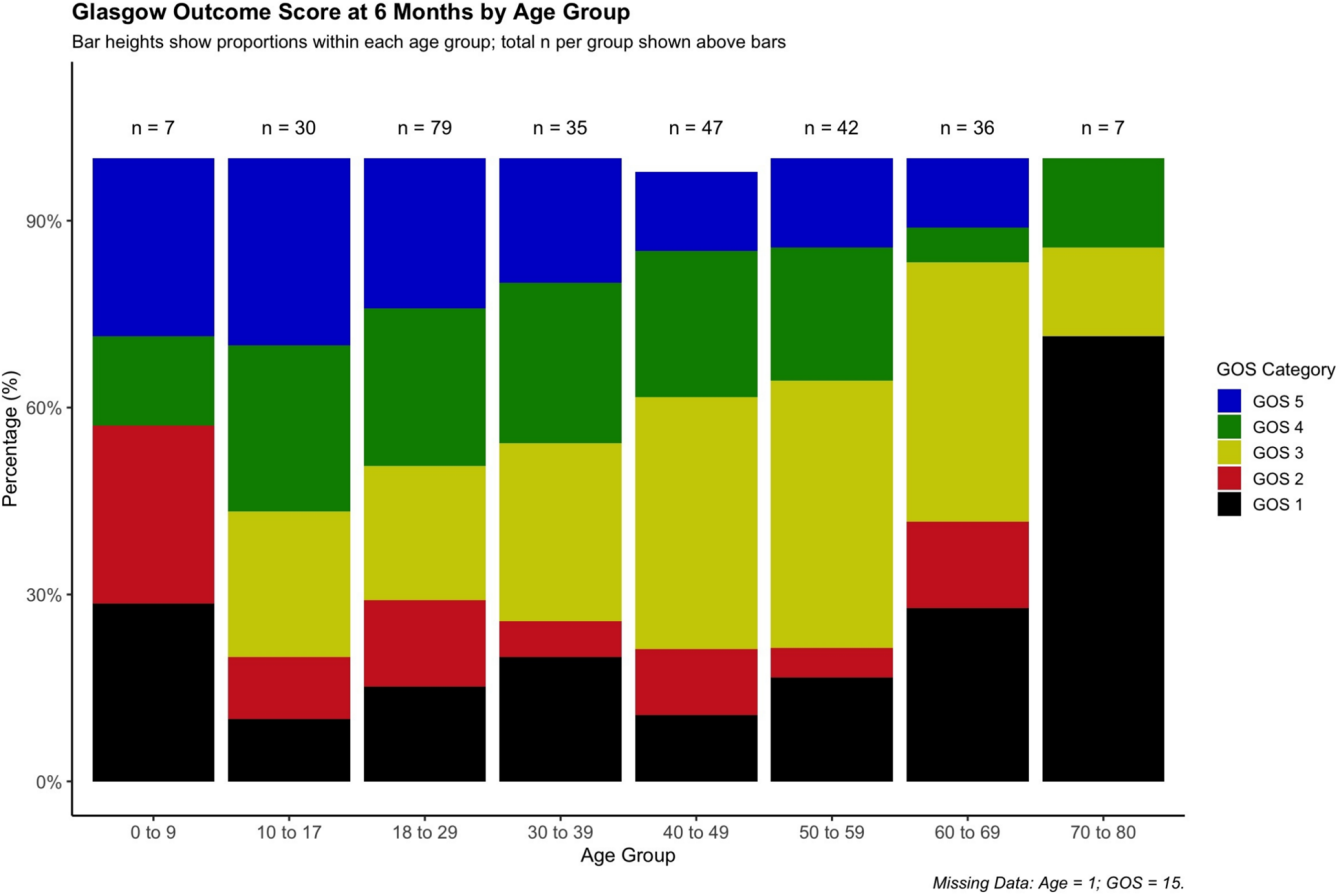
Age-Stratified Distribution of GOS at 6 Months post-injury

Surgical complications post-DC included re-operation due to haemorrhage in 10% of cases and complimentary bony decompression in 6% overall. Re-operation due to surgical-site infections occurred in 4%, re-operation due to subdural hygroma in 4%, and 12% required placement of a ventriculo-peritoneal shunt (Fig. 2).

There were modest inter-centre differences in treatment characteristics and outcomes. Umeå and Gothenburg performed DC slightly earlier (median day 1) than Stockholm and Uppsala (median day 2; *p* < 0.001). Although not statistically significant, the proportion of secondary DC varied, from 40% in Gothenburg to 64% in Umeå (*p* = 0.056). The type of DC also differed (*p* = 0.005), with bi-frontal procedures being more common in Gothenburg (20%) than in Stockholm (2%). Postoperative complications showed minor inter-centre variation, most particularly so in the rate of complimentary bony decompression (*p* = 0.041) and surgical-site infections (*p* = 0.006).

As shown in Supplementary Table 4, some differences were observed between adult and paediatric patients. Bi-frontal craniectomy was more frequently performed in paediatric cases (27% vs. 9%; *p* = 0.001). No other significant differences in indication or postoperative complications were identified.

### Impact of DC on mass effect

Radiologically, DC significantly reduced midline shift from a median of 6 mm (IQR 4–10) preoperatively to 2 mm (IQR 0–5) postoperatively (*p* < 0.001; Table 4). The proportion of patients with compressed or obliterated basal cisterns markedly decreased from 84% pre-DC to 45% post-DC (*p* < 0.001). The degree of midline shift and compression of the basal cisterns, both pre- and postoperatively, varied slightly between centres. Notably, Umeå showed a somewhat lower degree of preoperative midline shift, but the highest rate of basal cistern compression (95%) preoperatively.

**Table 4:** Mass effect before and after decompressive craniectomy – entire cohort and individual centres

**Radiological Variables**		All	Stockholm	Gothenburg	Uppsala	Umeå	***p*-value** **(centre)**
Midline shift (in mm), median (IQR)	Before	6 (4–10)	6 (4–9)	8 (4–12)	7 (5–11)	4 (0–8)	**< *0.001***
	After	2 (0–5)	3 (2–5)	4 (2–6)	2 (0–3)	0 (0–4)	**< *0.001***
	*P*-value	**< *0.001***	**< *0.001***	***0.002***	**< *0.001***	***0.007***	
Basal cisterns (open/compressed/obliterated), n (%)	Before	47/146/99 (16/50/34%)	28/32/17 (36/42/22%)	10/21/19 (20/42/38%)	5/58/25 (6/66/28%)	4/35/38 (5/46/49%)	**< *0.001***
	After	159/99/28 (55/35/10%)	44/25/5 (59/34/7%)	25/21/3 (51/43/6%)	79/6/3 (90/7/3%)	11/47/17 (15/63/22%)	**< *0.001***
	*P*-value	**< *0.001***	**< *0.001***	***0.042***	**< *0.001***	***0.011***	

**Missing Data:** Midline Shift Before DC = 6; Basal Cisterns Before DC = 7; Midline Shift After DC = 12; Basal Cisterns After DC = 13

Bold and italics represent statistical significance

### Functional outcome and prognostic factors

The median time post-injury to GOS assessment was 6 (IQR 5–8) months. At this follow-up, 60% of patients had recovered unfavourably and mortality was 11% (Table 2). Differences between centres were notable with Umeå reporting the highest rate of unfavourable outcome (72%), followed by Stockholm (61%), Uppsala (55%), and Gothenburg (49%). There were no statistically significant differences in either unfavourable outcome or mortality between adult and paediatric cohorts (Supplementary Table 1). An unfavourable outcome was observed in 64% of adults versus 46% of children with TBI (*p* = 0.098), and mortality was identical at 11% in both groups (*p* = 0.979).

The prognostic role of several variables is presented in Table 5. Older age, higher CCI scores, lower GCS M scores, obliterated basal cisterns before DC, and impaired pupillary reactivity were significantly associated with a higher rate of unfavourable outcomes in univariate logistic regression analyses (Table 5). Furthermore, in multivariable analyses (Table 6), older age (OR 1.04, 95% CI 1.03–1.08, *p* < 0.001), higher CCI scores (OR 1.75, 95% CI 1.09–3.23, *p* = 0.040), lower GCS (OR 0.68, 95% CI 0.55–0.86), *p* = 0.001), bilateral unreactive pupillary response (OR 9.09, 95% CI 2.63–50.00, *p* = 0.001), and obliterated basal cisterns (OR 3.70, 95% CI 1.39–11.11, *p* = 0.019) independently predicted a higher rate of unfavourable outcome. Moreover, mortality was significantly associated with bilateral unreactive pupillary response (OR 41.44, 95% CI 6.92–804.59, *p* < 0.001).

**Table 5:** Clinical outcome in relation to demography, injury severity, clinical course, and surgical aspects – Univariable logistic regression analysis

Variables	Mortality		Unfavourable Outcome	
	**OR (95% CI)**	***p-*value**	**OR (95% CI)**	***p-*value**
Age (years)	0.99 (0.96–1.02)	0.423	1.02 (1.01–1.04)	***0.005***
CCI Score	0.87 (0.38–1.46)	0.659	1.92 (1.27–3.23)	***0.006***
GCS M	0.63 (0.45–0.85)	***0.004***	0.72 (0.60–0.85)	**< *0.001***
Pupillary reaction, Normal	1.00 (Reference)	NA	1.00 (Reference)	NA
Unilateral unreactive	10.67 (1.53–211.68)	***0.04***	2.17 (1.10–4.35)	***0.029***
Bilateral unreactive	59.99 (10.87–112.47)	**< *0.001***	10.00 (3.57–50.00)	**< *0.001***
Marshall (1 point increase)	1.72 (0.99–3.78)	0.107	1.05 (0.83–1.33)	0.658
Barbiturates (yes)	1.03 (0.37–2.98)	0.961	1.01 (0.57–1.79)	0.979
Time from trauma to DC	0.81 (0.57–1.03)	0.157	0.94 (0.85–1.03)	0.174
Midline shift (mm) before DC	1.02 (0.91–1.13)	0.737	1.05 (0.99–1.12)	0.145
Basal Cisterns before DC (open)	1.00 (Reference)	NA	1.00 (Reference)	NA
Compressed	1.46 (0.22–28.57)	0.733	1.27 (0.57–2.78)	0.558
Obliterated	6.20 (1.11–1116.56)	0.089	2.78 (1.12–6.67)	***0.027***
Type of DC (Bilateral)	2.04 (0.44–7.05)	0.298	0.78 (0.32–1.92)	0.573
Indication for DC, primary	1.00 (Reference)	NA	1.00 (reference)	NA
Secondary	0.52 (0.18–1.44)	0.208	0.82 (0.46–1.45)	0.494
Hematoma evacuation (yes)	1.14 (0.35–5.16)	0.842	0.91 (0.44–1.82)	0.788
DC size (in cm^2^)	0.99 (0.96–1.01)	0.266	1.00 (0.99–1.01)	0.777

**Missing data**: Age = 1; GCS M = 47; CCI = 2; Pupillary Reaction = 14; Barbiturates = 11; Unfavourable Outcome = 15; Mortality = 15

Bold and italics represent statistical significance

*Abbreviations: CCI* Charlson Comorbidity Index, *GCS M* Glasgow Coma Scale Motor, *DC* Decompressive Craniectomy;

**Table 6:** Clinical outcome in relation to demography, injury severity, clinical course, and surgical aspects – multivariable logistic regression analysis

Variables	Mortality		Unfavourable Outcome	
	**OR (95% CI)**	***p-*value**	**OR (95% CI)**	***p-*value**
Age (years)	-	-	1.04 (1.03–1.08)	**< *0.001***
CCI score	-	-	1.75 (1.09–3.23)	***0.040***
GCS M	0.78 (0.51–1.14)	0.211	0.68 (0.55–0.86)	***0.001***
Pupillary reaction, Normal	1.0 (Reference)	NA	1.0 (Reference)	NA
Unilateral unreactive	6.67 (0.84–140.81)	0.110	1.33 (0.57–3.13)	0.501
Bilateral unreactive	41.44 (6.92–804.69)	**< *0.001***	9.09 (2.63–50.00)	***0.001***
Basal Cisterns before DC (open)	-	-	1.0 (Reference)	NA
Compressed	-	-	1.61 (0.65–4.00)	0.314
Obliterated	-	-	3.70(1.39–11.11)	***0.019***

**Mortality:** AIC = 89.68; *r*^2^ = 0.29; AUROC (95% CI) = 0.93 (0.89–0.96)

**Unfavourable Outcome**: AIC = 229.18; *r*^2^ = 0.22; AUROC (95% CI) = 0.79 (0.66–0.92)

**(-)** = not included in logistic regression model

Bold and italics represent statistical significance

*Abbreviations: GCS M* Glasgow Coma Scale Motor, *DC* Decompressive Craniectomy

## Discussion

Despite the advent of landmark RCTs DECRA and RESCUE-ICP [9, 11], this Swedish multicentre study shows that utilisation, indication, and timing of DC in TBI has remained largely unchanged over the past 15 years, suggesting a limited impact of these trials on clinical practice in high-resource settings. DC was mainly performed in young, severely injured patients, but with notable inter-centre variation in timing, indications, and surgical technique. Paediatric cases were rare but generally associated with favourable outcomes. Likewise, although few patients aged ≥ 60 years underwent DC, our findings indicate that favourable outcomes were achievable in selected cases. Overall, these results underscore the challenges of standardising care in a complex clinical setting and highlight the persistent gap between RCTs and real-world clinical practice.

### Temporal trends of DC in TBI management

The annual rate of DC per million inhabitants and year remained largely stable during the 15-year study period. Similarly, the proportion of primary vs. secondary DCs and timing post-injury showed no major temporal shifts. DECRA [9] reported worse outcomes with early, low-threshold DC, while RESCUE-ICP [11] demonstrated modest benefits when DC was performed at a late stage. Despite these influential studies published during the study period, the number of DC procedures was overall unchanged in the contemporary Swedish setting, suggesting limited impact on real-world practice, although we cannot rule out more granular potential shifts in timing, indication, or clinical thresholds for DC. However, there was a slight decline in the use of bi-frontal DC, possibly related to negative findings of this technique in the DECRA trial [9] or/and patient differences. RESCUE-ASDH [10] was published after the end of our study period and therefore could not have influenced clinical decision-making. Of note, in our trend analysis the DC rates were calculated per capita rather than in relation to the number of severe TBI cases, which may have changed over time. Although centre-specific data on severe TBI incidence are lacking, our clinical experience aligns with broader European trends [34], i.e., a decreasing number of young patients with severe TBI, and a relative increase in elderly patients. This suggests that the actual pool of potential DC candidates may have decreased over time, which should be considered when interpreting temporal trends.

### Patient selection, indication, and timing of DC – variation across centres and age groups

As expected, the overall DC cohort consisted of relatively young patients with high injury severity, as reflected by low motor scores (GCS M < 6), impaired pupillary reactivity, high Marshall grades, and degree of preoperative midline shift and basal cistern compression. This aligns with the prevailing understanding that DC is mainly considered in younger patients, and that refractory intracranial hypertension typically arises in the context of severe primary brain injury.

When comparing neurosurgical centres, age and sex distributions were similar; however, primary brain injury severity varied. Patients treated in Umeå and Stockholm presented with more severe initial injuries, including lower GCS M scores and more frequent pupillary abnormalities, which may reflect a tendency at these centres to offer DC mainly to patients with the most severe primary brain injuries. Differences in treatment approaches were also observed. For instance, evacuation of intracranial haematomas and use of barbiturate coma were less frequent in the DC cohort treated in Uppsala. These variations may reflect differences in underlying injury patterns, but also local treatment preferences, such as favouring DC over haematoma evacuation in cases with scattered contusions, or applying stricter criteria for initiating barbiturate coma or usage of osmotherapy [8]

Most DC procedures were performed within 72 h of trauma, with slightly earlier timing in Gothenburg and Umeå (median day 1) compared to Stockholm and Uppsala (median day 2). Primary and secondary DC were generally evenly distributed across centres, although Gothenburg had a predominance of primary DC (60%), and Umeå of secondary DC (64%). The predominance of secondary DC despite early timing in Umeå is notable and suggests a deliberate strategy to confirm intracranial hypertension through ICP monitoring, rather than relying solely on radiological or clinical signs, before proceeding with DC, even though the decision often appears to have been made rapidly. Regarding surgical technique, bi-frontal DC was rarely performed overall and, particularly, in Stockholm it accounted for only 2%, while hemicraniectomy was the main DC type. In contrast, both Gothenburg and Umeå applied bi-frontal DC in approximately 20% of cases. These differences may reflect variations in underlying brain injury patterns; bi-frontal DC is often preferred in cases of diffuse, bilateral brain swelling with compressed basal cisterns, whereas hemi-DC is typically more appropriate when the brain injury is lateralized and there is significant midline shift. This pattern was seen in Umeå, which showed a lower rate of midline shift but a higher degree of basal cistern compression on preoperative CT compared to other centres. Another possible factor is the interpretation of key RCTs: DECRA primarily investigated bi-frontal DC and showed worse outcomes compared to medical management [9], whereas RESCUE-ICP mainly involved hemi-DC and demonstrated a modest benefit [11]. While the surgical approach was by no means the only difference between the trials, it represents a key distinction that may have influenced surgical preferences at different centres. Regardless of surgical approach, the bone flap size was generally adequate and consistent across all centres, with nearly all procedures involving an area close to 100 cm^2^.

DC rates per capita were largely comparable across centres, except in Umeå, which had nearly double the rate. As the northernmost centre, Umeå covers the largest geographic area but serves the smallest population. This discrepancy may reflect a combination of factors, including a different panorama of TBI patients, longer transport times leading to worsened secondary injury and higher risk of ICP crises, as well as possible differences in clinical thresholds for proceeding with DC [12, 13, 15]. Paediatric DC was uncommon, comprising only 37 patients (12% of the cohort), which was expected given that moderate-to-severe TBI is significantly more prevalent among adults than children in European populations [35, 36]. Notably, the vast majority of paediatric cases were adolescents, with only seven children under the age of 10. Compared to adults, paediatric patients were more often female (38% vs. 22%), less frequently required haematoma evacuation (70% vs. 82%), and more commonly received bi-frontal DC (9% vs. 27%). These findings likely reflect the tendency for younger patients to develop diffuse brain swelling and raised ICP in the absence of major haemorrhagic mass lesions. An additional noteworthy finding regarding age was, at the other end of the spectrum, that several patients aged 70 years and older were offered DC. These were likely highly selected cases with a perceived reasonable chance of recovery, and serves as an important observation in light of the shifting TBI epidemiology in high-income countries [34], from young individuals involved in road traffic accidents to elderly patients sustaining falls.

### DC- treatment effects, complications, and long-term outcome

The short-term effects of DC were generally good. As expected, the procedure significantly reduced intracranial mass effect, evidenced by a decreased midline shift and improved basal cistern status postoperatively. The overall rate of postoperative complications was relatively low. The most common complication was postoperative haemorrhage requiring re-operation, occurring in approximately 10% of cases, which is consistent with the literature [37]. This complication rate is higher than after DC for other brain injury conditions such as aneurysmal subarachnoid haemorrhage [38]. Several factors likely contribute; first, TBI, per se, may elicit significant coagulopathy-issues [39]; second, DC results in a rapid reduction in ICP, which diminishes the tamponade effect on existing haemorrhagic lesions [14, 40]; and third, some haemorrhagic progression may occur independently of surgery, as part of the natural evolution of traumatic injury [40]. Re-operations due to decompression expansion, subdural hygroma, or surgical-site infection were infrequent, each affecting approximately 5% of patients, rates that are relatively low compared to previous systematic reviews [38, 41].

In terms of long-term functional outcomes, 60% (64% for adults only) of patients in our cohort recovered unfavourably and 11% were deceased at 6 months (only in adults). These results are more encouraging than those reported in the major RCTs, where unfavourable outcomes were 73% in RESCUE-ICP [11], 70% in DECRA [9], and 72% in RESCUE-ASDH [19], with corresponding mortality rates of 27%, 19%, and 28%, respectively. Furthermore, expected rates of unfavourable outcome and mortality for adult patients in this cohort were estimated using the IMPACT core prognostic model, yielding predicted rates of 47% (IQR 32–69) and 31% (IQR 18–47), respectively. These estimates indicate a somewhat higher rate of unfavourable outcome, but a lower mortality rate, compared with the observed cohort. However, these comparisons should be interpreted with caution, as the IMPACT core model is limited to coarse demographic variables and markers of primary brain injury, and does not account for subsequent clinical events or secondary brain insults that may have occurred prior to or during the course of care and contributed to the need for decompressive craniectomy in this highly selected cohort.

Our results likely reflect real-world clinical practice, where DC is used selectively and guided by individualised clinical judgement, targeting patients considered most likely to benefit and at a time-point deemed optimal. In contrast, RCTs are constrained by strict inclusion and exclusion criteria and often examine DC within predefined contexts (e.g., primary vs. secondary DC, early vs. late intervention), which may not reflect the nuanced and complex decision-making encountered in everyday care and may not be optimal to achieve the best outcomes for the patients. This discrepancy in patient selection, timing, and context likely contributes to the differences in outcomes, and also underscores the inherent difficulty in designing phase III trials for last-resort interventions within a complex management protocol and a heterogeneous disease such as TBI. Although we lacked access to detailed ICP data and therefore could not retrospectively assess eligibility for DECRA [9] or RESCUE-ICP [11] we would speculate that most patients receiving secondary DC in our cohort would have met the low-threshold criteria for DECRA, but with relatively few eligible for the high-threshold RESCUE-ICP trial [9, 11].

Altogether, these findings support the notion that real-world use of DC in modern NIC units may result in better outcomes than those reported in RCTs with narrow inclusion criteria. This may, at least in some extent, also explain why such trials have had limited impact on clinical practice. At the same time, reliance on individualised judgement presents a significant barrier to advancing the field. While such an approach may optimise care for the individual patient, it inevitably leads to inconsistent application across centres and clinicians, and a lack of standardised care. Consequently, it remains challenging to systematically identify which patients truly benefit from DC, thereby limiting our ability to generate high-quality evidence that can guide future practice and improve clinical guidelines.

Furthermore, the 60% rate of unfavourable outcome found in this cohort of DC treated TBI patients was lower than what is typically observed in other acute brain injuries such as aneurysmal subarachnoid haemorrhage (around 80–90% [31, 42, 43]) and malignant middle cerebral artery infarction (around 75–85% [31, 42, 43]). In these latter conditions, cytotoxic brain injury from large territorial infarctions is common, whereas in TBI, the oedema is more often vasogenic, involving potentially salvageable brain tissue [44]. This difference in pathophysiology may partly explain the more favourable outcomes seen in TBI patients experiencing ICP crises requiring DC compared to other forms of acute brain injury.

### Outcome prediction

Our findings largely corroborate that the predictive factors, chronological age, primary brain injury severity (GCS M and pupillary reactivity), and signs of mass effect (basal cistern compression), suggested by the IMPACT [45] and CRASH [46] studies, remain generally valid even in the sub-cohort of TBI. Co-morbidities indicating biological age, as measured by the CCI [31], also showed relevance in prediction of unfavourable outcome.

Adult patients demonstrated slightly more unfavourable recovery than paediatric ones (64% vs. 46%, p = 0.098), potentially due to greater neuroplasticity and regenerative capacity of the latter [47, 48]. Another contributing factor may be that children’s lower baseline intracranial compliance is likely to result in elevated ICP from relatively milder primary brain injuries. In contrast, adults, particularly the elderly, often have more intracranial reserve due to cerebral atrophy, potentially masking pressure elevations until more extensive injury has developed. This may allow for better recovery in paediatric patients once the ICP crisis is resolved. It is nevertheless notable that many patients aged 60 and older, including those in their 70 s, experienced favourable recovery.

At the other end of the age spectrum, patients aged 70 years and older are often not considered candidates for aggressive interventions such as DC in the context of TBI, but also aneurysmal subarachnoid haemorrhage, and malignant middle cerebral artery infarction. Nonetheless, functional recovery was observed in some of the older patients in our cohort. This likely reflects a highly selected subgroup, treated based on favourable pre-morbid status and a perceived potential for meaningful outcome, rather than representing older TBI population in general. Still, these findings underscore that favourable outcome is achievable in selected cases of senior TBI patients.

Otherwise, few variables directly related to the surgical procedure itself, such as timing, indication (primary vs. secondary), type (hemicraniectomy vs. bi-frontal), or bone flap size, were significantly associated with outcome. This suggests that patient- and injury-related factors may play a more decisive role in prognosis than procedural details within the DC-treated population. Additionally, there is a need to further explore underlying genetic variation, the neuroanatomical localisation of injury, and cellular patterns of damage as prognostic factors [49]*.*

### Methodological considerations

This study has several important strengths. It is a large, multicentre cohort of DC-treated TBI patients in a high-income setting with consistent access to NIC. The 15-year study period enabled evaluation of temporal trends, while inclusion of both adult and paediatric patients allowed for age-stratified comparisons. The use of detailed clinical, radiological, and surgical data across multiple centres enhances generalisability and enables meaningful assessment of inter-centre variation. Additionally, structured 6-month outcome data provide valuable insight into real-world effectiveness.

However, several limitations must be acknowledged. First, the retrospective observational study design introduces potential selection bias and residual confounding, limiting causal inference. Second, differences in treatment protocols and clinical practices across centres may have introduced heterogeneity, complicating interpretation of pooled outcomes. Third, the absence of a matched control group or randomisation precludes direct comparison with alternative treatment strategies, thereby limiting conclusions about the absolute efficacy. Fourth, outcome assessments were conducted retrospectively, with possible variability despite efforts to standardise grading. Fifth, we did not have access to long-term follow-up beyond six months, potentially missing late recovery or complications. Sixth, the absence of Injury Severity Score data limits our ability to compare systemic injury severity across patient groups. Lastly, the lack of detailed ICP data limits our ability to compare our findings to trial populations such as those in DECRA and RESCUE-ICP [9, 11].

## Conclusions

This 15-year multicentre cohort study provides an in-depth overview of DC practices for TBI in Sweden. Despite the publication of landmark RCTs during this period, DC use remained relatively stable, suggesting limited impact on real-word practice. There were substantial inter-centre differences in indications, timing, surgical technique, and outcomes, reflecting the complexity and heterogeneity of TBI care. DC was primarily performed in young patients with severe injuries, but also in a few highly selected senior adults. Paediatric patients, though few, showed slightly better outcomes and different surgical profiles. Overall, DC effectively reduced radiological mass effect, with slightly less complications and more favourable functional outcomes than those reported in major RCTs. Notably, challenges in designing RCTs to determine the optimal role of DC in TBI make additional trials unlikely in the foreseeable future, while future prospective studies should aim to bridge the gap between trial-based evidence and clinical reality.

## Supplementary Information

Supplementary Material 1.

## Data Availability

No datasets were generated or analysed during the current study.
