# Supplementary material for: Decompressive craniectomy in traumatic brain injury: insights from a 15-year multicentre cohort in Sweden
Source: Scand J Trauma Resusc Emerg Med. 2026 Feb 26;34:51. doi: 10.1186/s13049-026-01585-6 (PMC12954973; doi:10.1186/s13049-026-01585-6)
Supplement: Supplementary file 1 — Supplementary Material 1. [file 13049_2026_1585_MOESM1_ESM.docx]

**Supplementary Table 1.** Demographics, admission variables, clinical course, and functional outcome for Entire Cohort – Comparison of adult vs paediatric populations.

| **Variables** | **Entire Cohort** | | ***P*-value** |
| --- | --- | --- | --- |
|  | **Adult** | **Paediatric** |  |
| Patients, n (%) | 261 (88%) | 37 (12%) | N/A |
| Age (years), median (IQR) | 42 (26-53) | 15 (12-16) | N/A |
| Sex (male/female), n (%) | 204/57 (78/22%) | 23/14 (62/38%) | 0.053 |
| CCI Score, n (%) | | | |
| 0 | 65 (25%) | 34 (92%) | ***<0.001*** |
| ≥ 1 | 194 (75%) | 3 (8%) |  |
| GCS M at admission, median (IQR) | 4 (2-5) | 4 (1-5) | 0.669 |
| Pupillary Reaction at admission, n (%) | | | |
| *Normal, n (%)* | 129 (52%) | 18 (51%) | 0.792 |
| *1 Unreactive, n (%)* | 67 (27%) | 8 (23%) |  |
| *2 Unreactive, n (%)* | 53 (21%) | 9 (26%) |  |
| Marshall Classification, n (%) | | | |
| *I-II, n (%)* | 28 (11%) | 6 (16%) | 0.069 |
| *III-IV, n (%)* | 18 (7%) | 6 (16%) |  |
| *V-VI, n (%)* | 215 (82%) | 25 (68%) |  |
| ICP-monitoring, n (%) | | | |
| *No ICP-monitoring* | 2 (1%) | 1 (3%) | 0.296 |
| *EVD* | 39 (15%) | 3 (8%) |  |
| *Intraparenchymal* | 136 (52%) | 17 (46%) |  |
| *Both* | 84 (32%) | 16 (43%) |  |
| Intracranial hematoma Evacuation (yes), n (%) | 214 (82%) | 26 (70%) | 0.092 |
| Barbiturates (yes), n (%) | 138 (55%) | 21 (60%) | 0.687 |
| Unfavourable Outcome*, n (%) | 167 (64%) | 17 (46%) | 0.098 |
| Mortality, n (%) | 29 (11%) | 4 (11%) | 0.979 |
| **Missing data:** Age = 1; CCI = 2; GCS M = 47; Pupillary Reaction = 14; Barbiturates = 11; Unfavourable Outcome = 15; Mortality = 15.  **Abbreviations:** GCS M = Glasgow Coma Scale Motor; CCI = Charlson Co-morbidity Index; GOS = Glasgow Outcome Scale  ***** Unfavourable Outcome = GOS 1-3  Bold and italics represent statistical significance | | | |

Expected values for mortality and unfavourable outcome for adult patients in this cohort were calculated as a median of 31% (IQR=18-47, p<0.001) and 47% (IQR=32-69, p<0.001) respectively according to IMPACT core prognostic model.

**Supplementary Table 2.** Demographics, Admission Variables, and Outcomes in Exclusively Adult Patients – Comparison between centres

| **Variables,** | **Stockholm** | **Gothenburg** | **Uppsala** | **Umeå** | ***p*-value** |
| --- | --- | --- | --- | --- | --- |
| Patients, n (%) | 70 (27%) | 47 (18%) | 77 (30%) | 67 (25%) | N/A |
| Age (years), median (IQR) | 34 (25-49) | 43 (26-53) | 44 (29-55) | 43 (26-59) | 0.226 |
| Sex (male/female), n (%) | 56/14 (80/20%) | 38/9 (81/19%) | 58/19 (75/25%) | 52/15 (78/22%) | 0.870 |
| *CCI Score, n (%)* | | | | | |
| 0 | 54 (79%) | 28 (60%) | 63 (82%) | 49 (73%) | ***0.033*** |
| ≥ 1 | 14 (21%) | 19 (40%) | 14 (18%) | 18 (27%) |  |
| GCS M at admission, median (IQR) | 3 (1-5) | 6 (5-6) | 5 (3-5) | 3 (2-5) | ***<0.001*** |
| *Pupillary Reaction at admission, n (%)* | | | | | |
| *Normal, n (%)* | 28 (42%) | 22 (47%) | 54 (70%) | 25 (43%) | ***<0.001*** |
| *1 Unreactive, n (%)* | 26 (39%) | 11 (23%) | 15 (20%) | 15 (26%) |  |
| *2 Unreactive, n (%)* | 13 (19%) | 14 (30%) | 8 (10%) | 18 (31%) |  |
| *Marshall Classification, n (%)* | | | | | |
| *I-II, n (%)* | 1 (1%) | 0 (0%) | 23 (30%) | 4 (6%) | ***<0.001*** |
| *III-IV, n (%)* | 4 (6%) | 0 (0%) | 6 (8%) | 8 (12%) |  |
| *V-VI, n (%)* | 65 (93%) | 47 (100%) | 48 (62%) | 55 (82%) |  |
| *ICP-monitoring, n (%)* | | | | | |
| *No ICP-monitoring* | 1 (1%) | 1 (2%) | 0 (0%) | 0 (0%) | ***<0.001*** |
| *EVD* | 19 (27%) | 9 (19%) | 6 (8%) | 5 (8%) |  |
| *Intraparenchymal* | 27 (39%) | 27 (57%) | 51 (66%) | 31(46%) |  |
| *Both* | 23 (33%) | 10 (22%) | 20 (26%) | 31(46%) |  |
| Intracranial hematoma Evacuation (yes), n (%) | 65 (93%) | 41 (87%) | 59 (77%) | 49 (73%) | ***0.009*** |
| Barbiturates (yes), n (%) | 37 (53%) | 30 (65%) | 25 (32%) | 46 (78%) | ***<0.001*** |
| Unfavourable Outcome*, n (%) | 43 (58%) | 24 (50%) | 48 (60%) | 52 (76%) | ***0.008*** |
| Mortality, n (%) | 8 (11%) | 4 (9%) | 4 (5%) | 13 (19%) | 0.052 |
| **Missing data:** CCI = 2; GCS M = 42; Pupillary Reaction = 12; Barbiturates = 9; Unfavourable Outcome = 15; Mortality 15.  **Abbreviations:** GCS M = Glasgow Coma Scale Motor; CCI = Charlson Co-morbidity Index; GOS = Glasgow Outcome Scale  ***** Unfavourable Outcome = GOS 1-3  Bold and italics represent statistical significance | | | | | |

**Supplementary Table 3.** Demographics, Admission Variables, and Outcomes in Exclusively Paediatric Patients – Comparison between centres

| **Variables,** | **Stockholm** | **Gothenburg** | **Uppsala** | **Umeå** | ***p*-value** |
| --- | --- | --- | --- | --- | --- |
| Patients, n (%) | 11 (30%) | 3 (8%) | 12 (32%) | 11 (30%) | NA |
| Age (years), median (IQR) | 16 (14-17) | 14 (8-16) | 15 (13-16) | 15 (13-16) | 0.266 |
| Sex (male/female), n (%) | 7/4 (64/36%) | 2/1 (67/33%) | 9/3 (75/25%) | 5/6 (45/55%) | 0.547 |
| CCI Score, n (%) | | | | | |
| 0 | 11 (100%) | 2 (67%) | 12 (100%) | 9 (82%) | 0.115 |
| ≥ 1 | 0 (0%) | 1 (33%) | 0 (0%) | 2 (18%) |  |
| GCS M at admission, median (IQR) | 1 (1-3) | 6 (6-6) | 5 (4-5) | 3 (1-5) | ***0.022*** |
| Pupillary Reaction at admission, n (%) | | | | | |
| *Normal, n (%)* | 2 (20%) | 1 (33%) | 8 (73%) | 7 (64%) | ***<0.001*** |
| *1 Unreactive, n (%)* | 4 (40%) | 0 (0%) | 1 (9%) | 3 (27%) |  |
| *2 Unreactive, n (%)* | 4 (40%) | 2 (67%) | 2 (18%) | 1 (9%) |  |
| Marshall Classification, n (%) | | | | | |
| *I-II, n (%)* | 1 (9%) | 0 (0%) | 4 (33%) | 1 (9%) | 0.246 |
| *III-IV, n (%)* | 1 (9%) | 0 (0%) | 4 (33%) | 1 (9%) |  |
| *V-VI, n (%)* | 9 (82%) | 3 (100%) | 4 (33%) | 9 (82%) |  |
| ICP-monitoring, n (%) | | | | | |
| *No ICP-monitoring* | 1 (9%) | 0 (0%) | 0 (0%) | 0 (0%) | ***<0.001*** |
| *EVD* | 1 (9%) | 0 (0%) | 1 (8%) | 1 (9%) |  |
| *Intraparenchymal* | 6 (55%) | 1 (33%) | 6 (50%) | 4 (36%) |  |
| *Both* | 3 (27%) | 2 (67%) | 5 (42%) | 6 (55%) |  |
| Intracranial hematoma Evacuation (yes), n (%) | 9 (82%) | 2 (67%) | 6 (50%) | 9 (82%) | 0.286 |
| Barbiturates (yes), n (%) | 6 (55%) | 3 (100%) | 4 (33%) | 8 (89%) | ***0.032*** |
| Unfavourable Outcome*, n (%) | 8 (73%) | 1 (33%) | 3 (25%) | 4 (45%) | 0.139 |
| Mortality, n (%) | 4 (36%) | 0 (0%) | 0 (0%) | 0 (0%) | ***0.014*** |
| **Missing data:** GCS M = 5; CCI = 0; Pupillary Reaction = 2; Barbiturates = 2.  **Abbreviations:** GCS M = Glasgow Coma Scale Motor; CCI = Charlson Co-morbidity Index; GOS = Glasgow Outcome Scale  ***** Unfavourable Outcome = GOS 1-3  Bold and italics represent statistical significance | | | | | |

**Supplementary table 4.** DC surgery: timing, indication, and complications – adult vs paediatric populations

| Variables | Entire Cohort | | p-value |
| --- | --- | --- | --- |
|  | Adult | Paediatric |  |
| Time from trauma to DC (days), median (IQR) | 1 (1-3) | 1 (1-2) | 0.575 |
| Indication for DC (primary/secondary), n (%) | 121/140 (46/54%) | 21/16 (57/43%) | 0.236 |
| Type of DC (hemi/bifrontal), n (%) | 237/24 (91/9%) | 27/10 (73/27%) | ***0.001*** |
| DC size (in cm^2^), median (IQR) | 96.4 (83.8-109.0) | 101.5 (84.5-122.6) | 0.399 |
| Reintervention due to Post-DC bleeding (yes), n (%) | 26 (10%) | 3 (8%) | 0.721 |
| Post-DC extension of bony decompression, n (%) | 16 (6%) | 1 (3%) | 0.395 |
| Post-DC surgical site-infection, n (%) | 10 (4%) | 1 (3%) | 0.733 |
| Post-DC subdural hygroma, n (%) | 9 (3%) | 3 (8%) | 0.177 |
| Post-DC VP-shunt, n (%) | 31 (12%) | 5 (14%) | 0.775 |
| **Missing Data:** DC Size (in cm^2^) = 11  **Abbreviations:** DC = Decompressive Craniectomy; VP-shunt = Ventriculoperitoneal shunt.  ***** Progress of hematoma defined as increase in volume > 10 ml  Bold and italics represent statistical significance | | | |

**Supplementary table 5.** DC surgery: timing, indication, and complications in Exclusively Adult Patients – Comparison between centres

| Variables,  Adult/Paediatric | Stockholm | Gothenburg | Uppsala | Umeå | p-value |
| --- | --- | --- | --- | --- | --- |
| Time from trauma to DC (days), median (IQR) | 1 (1-2) | 1 (1-2) | 2 (1-4) | 1 (1-2) | ***0.016*** |
| Indication for DC (primary/secondary), n (%) | 31/39 (44/56%) | 27/20 (57/43%) | 39/38 (51/49%) | 24/43 (36/64%) | 0.111 |
| Type of DC (hemi/bifrontal), n (%) | 70/0 (100/0%) | 38/9 (81/19%) | 70/7 (91/9%) | 59/8 (88/12%) | **0.004** |
| DC size (in cm^2^), median (IQR) | 96.6 (87.6 – 109.7) | 92.0 (74.3-107.0) | 98.0 (85.6-113.7) | 94.7 (83.6-106.3) | 0.492 |
| Reintervention due to Post-DC bleeding (yes), n (%) | 6 (9%) | 8 (17%) | 5 (6%) | 7 (10%) | 0.282 |
| Post-DC extension of bony decompression, n (%) | 0 (0%) | 5 (11%) | 5 (6%) | 6 (9%) | 0.066 |
| Post-DC surgical site-infection, n (%) | 1 (1%) | 6 (13%) | 3 (4%) | 0 (0%) | ***0.002*** |
| Post-DC subdural hygroma, n (%) | 3 (4%) | 1 (2%) | 2 (3%) | 3 (4%) | 0.853 |
| Post-DC VP-shunt, n (%) | 8 (11%) | 8 (17%) | 8 (10%) | 6 (9%) | 0.410 |
| **Missing Data:** DC Size (in cm^2^) = 10  **Abbreviations:** DC = Decompressive Craniectomy; VP-shunt = Ventriculoperitoneal shunt.  ***** Progress of hematoma defined as increase in volume > 10 ml  Bold and italics represent statistical significance | | | | | |

**Supplementary table 6.** DC surgery: timing, indication, and complications in Exclusively Paediatric Patients – Comparison between centres

| Variables,  Adult/Paediatric | Stockholm | Gothenburg | Uppsala | Umeå | p-value |
| --- | --- | --- | --- | --- | --- |
| Time from trauma to DC (days), median (IQR) | 1 (1-1) | 1 (1-2) | 2 (1-3) | 2 (1-4) | ***0.022*** |
| Indication for DC (primary/secondary), n (%) | 9/2 (82/18%) | 3/0 (100/0%) | 5/7 (42/58%) | 4/7 (36/64%) | ***0.044*** |
| Type of DC (hemi/bifrontal), n (%) | 9/2 (82/18%) | 2/1 (67/33%) | 10/2 (83/17%) | 6/5 (55/45%) | 0.385 |
| DC size (in cm^2^), median (IQR) | 98.7 (93.1-104.4) | 45.0 (41.0-92.5) | 111.4 (100.5-122.6) | 98.4 (82.0-171.6) | 0.347 |
| Reintervention due to Post-DC bleeding (yes), n (%) | 2 (18%) | 0 (0%) | 0 (0%) | 0 (0%) | 0.434 |
| Post-DC extension of bony decompression, n (%) | 0 (0%) | 0 (0%) | 0 (0%) | 1 (9%) | 0.488 |
| Post-DC surgical site-infection, n (%) | 0 (0%) | 0 (0%) | 0 (0%) | 1 (9%) | 0.232 |
| Post-DC subdural hygroma, n (%) | 0 (0%) | 0 (0%) | 2 (17%) | 1 (9%) | 0.478 |
| Post-DC VP-shunt, n (%) | 0 (0%) | 0 (0%) | 4 (33%) | 1 (9%) | 0.093 |
| **Missing Data:** DC Size (in cm^2^) = 1  **Abbreviations:** DC = Decompressive Craniectomy; VP-shunt = Ventriculoperitoneal shunt.  ***** Progress of hematoma defined as increase in volume > 10 ml  Bold and italics represent statistical significance | | | | | |
